# Dynamics of Students’ Career Choice: a Conceptual Framework–Based Qualitative Analysis Focusing on Primary Care

**DOI:** 10.1007/s11606-023-08567-9

**Published:** 2023-12-15

**Authors:** Eva Pfarrwaller, Hubert Maisonneuve, Camille Laurent, Milena Abbiati, Johanna Sommer, Anne Baroffio, Dagmar M. Haller

**Affiliations:** 1 University Institute for Primary Care, Faculty of Medicine, University of Geneva, Geneva, Switzerland; 2 University College of General Medicine, Faculty of Medicine, University Claude Bernard Lyon 1, Lyon, France; 3 Unit of Development and Research in Medical Education, Faculty of Medicine, University of Geneva, Geneva, Switzerland

**Keywords:** career choice, undergraduate medical education, primary care physicians, workforce, qualitative research

## Abstract

**Background:**

Increasing primary care’s attractiveness as a career choice is an important task of socially accountable medical schools. Research has broadly studied influences on medical students’ career choice. However, a deeper understanding of the processes behind career decision-making could support medical schools in their efforts to promote primary care careers.

**Objective:**

To explore the dynamics of career choice during medical school with a focus on primary care, based on a previously developed conceptual framework.

**Approach:**

Qualitative study using a phenomenological, inductive-deductive approach

**Design and Participants:**

Individual interviews were conducted from May 2019 to January 2020 with 14 first-year postgraduate trainee physicians, graduates of the Faculty of Medicine in Geneva, Switzerland, purposively sampled based on their interest in primary care during undergraduate studies. The interview guide was developed to elicit narratives about career-related decision-making. Two authors coded the transcripts. Thematic analysis alternated with data collection until thematic saturation was reached. Emerging themes were discussed and refined within the research team.

**Key Results:**

Two main themes emerged: (1) developing professional identity, expressed as a changing professional image from unprecise and idealistic to concrete and realistic; priorities changed from content-based to lifestyle-based preferences; (2) individual trajectories of career-related decision-making, determined by different stages of refining professional interests; students navigated this process by employing various strategies, ranging from active exploration to passive behaviors.

**Conclusions:**

This study’s narrative approach illustrates the dynamic nature of career choice and refines elements of a conceptual framework previously developed by the authors. Its findings underline the importance of exploration, for which personal experiences and observations of physicians’ work are crucial. To advance efforts to make primary care a more attractive career, students must be sufficiently exposed to primary care in a safe and individualized environment and should be supported in all stages of their career choice process.

**Supplementary Information:**

The online version contains supplementary material available at 10.1007/s11606-023-08567-9.

## BACKGROUND

Health systems that heavily rely on primary care improve population health and are more cost-effective.^1,2^ Despite clear evidence in favor of this statement, primary care workforce shortages limit the translation of this evidence in many countries.^3^ Many medical schools endorse the responsibility to be socially accountable to the community they serve, which includes making primary care a more attractive career choice for students.^4^ Research has identified numerous factors influencing career choice.^5,6^ We proposed a revised conceptual framework of primary care career choice integrating these factors to provide avenues for research and medical education aiming to strengthen primary care at the undergraduate level.^7^ Although this framework was based on published research and models of career choice, it remained largely theoretical. The present study therefore aimed to confront this framework to real-life experiences by exploring students’ career decision-making.

Past research has focused on specific predictors, contributing knowledge about *what* determines career choice.^6^ More recently, qualitative studies have enriched our understanding of *how* and *why* choices are made, highlighting the importance of clinical exposure, students’ need for work-life balance, or the role of the hidden curriculum.^8–11^ Students’ career preferences evolve between matriculation and graduation, but we still know little about these changes in individual students.^12^ The aim of our conceptual framework was to provide a theoretical basis to support our mission of making primary care a more attractive career choice during undergraduate medical education. It represented career choice in terms of interest in primary care and individual decision-making processes, assuming that students could be grouped into distinct trajectories based on their initial level of commitment to primary care.^13^ Please refer to Supplemental Digital Appendix 1 for a detailed description of the framework.

Recent research found that students’ career intentions vary on a continuum from undecidedness to firm commitment, and that external influences might be the main drivers of these changes.^14^ Qualitative studies contributed insights into this process and suggested that students iteratively reevaluate career preferences.^15–17^ However, a deeper understanding of the career choice process focusing on changes over time could enrich our knowledge base for medical schools’ efforts to make primary care more attractive.^17^

Our study’s aim was to qualitatively explore the dynamics of career choice during undergraduate medical education with a focus on primary care, using our conceptual framework as a theoretical basis and critically confronting it to the findings. In line with our mission, we specifically wanted to gain more insight into actionable factors that could inform a primary care–promoting undergraduate curriculum.

In this article, we address the following research questions:

- How do career choices emerge during medical school, and can patterns of decision-making be identified?
- What strategies and actions do students apply to advance in their career decision-making process?

## METHODS

### Approach and Design

Former students of the Faculty of Medicine in Geneva, Switzerland (see Text Box 1 for context) were interviewed during their first postgraduate training year. Narratives about career decision-making were analyzed thematically, adopting a phenomenological approach.^18^ Our study was guided by a constructivist paradigm, assuming that career choice is a subjective process.^19^

**Text Box 1** Academic and Medical Context of a Qualitative Study on Medical Students’ Primary Care Career Choice Processes

**Table:** 

Undergraduate medical training
• Year 1: Pre-selection year (mostly basic sciences) • Years 2 and 3: Pre-clinical years (incl. 4 half-days in a primary care practice) • Years 4 and 5: Clinical clerkships (mandatory), mostly hospital-based (incl. 8 half-days in a primary care practice) • Year 6: 10 months of mostly freely chosen clinical clerkships (incl. 1 month in a primary care practice) Postgraduate medical training • All graduates have unrestricted choice of any specialty regardless of examination results or other criteria. • To practice medicine independently, it is mandatory to acquire a federal specialist title in one of the 45 disciplines by completing the minimal requirements as stated by the specialty’s professional organization. • Training is mostly organized by residents themselves (except for a few formal training programs): Residents choose where to apply for training positions and structure their training according to the specialty’s requirements (such as minimal time, time to be spent in university hospitals, time to be spent in outpatient practice). There are no uniform criteria for hospitals to select their residents. • The specialist titles in general internal medicine and pediatrics require a minimal training duration of 5 years. Training in primary care practices is possible for a limited time, but not mandatory. Hence, future primary care physicians are mostly trained in hospital contexts (in- or outpatient care). Definition of primary care • No specific primary care specialty (such as family medicine) • Primary care physicians are general internists or pediatricians practicing in an ambulatory setting according to the principles of family medicine/general practice (i.e., serving as first point of care for most health problems for an unselected patient population).

### Participants

The sample consisted of physicians who had graduated in 2018 and had participated, during their undergraduate studies, in a cohort study exploring academic performance and career choices.^20,21^ We applied a purposive sampling strategy within this cohort to select participants with several degrees of interest in primary care. We hypothesized that consistently uninterested students would be difficult to attract to primary care. Thus, and in line with our aim to identify actionable factors related to primary care career choice, we purposefully chose to include only participants who had expressed an interest in primary care during their studies, regardless of their career choice at graduation. We expected this group to contribute the most relevant insights, not only about choosing primary care as a career, but also about the processes that led them to turn away from primary care during their studies. Thirty-three potential participants were invited by e-mail in three iterative recruitment rounds, of which 14 were interviewed (see Supplemental Digital Appendix 2 for sampling details). Participants provided written consent before the interview.

### Data Collection and Analysis

Semi-structured individual interviews were conducted in-person between May 2019 and January 2020 by the first author at quiet locations suiting the participants. They were conducted in French, audio-recorded, and transcribed verbatim; identifying data were anonymized. The interview guide was based on our conceptual framework and is provided in Supplemental Digital Appendix 3. Participants were first asked about their current professional activity and how they had arrived there; this usually elicited a rich narrative about career-related decisions. Probing questions ensured coverage of predefined topics and all periods of medical school. Primary care–related questions were raised if they had not arisen spontaneously. Participants were aware of the study topic (career choice) and did not know the interviewer but knew that she worked at a primary care institute. They received a bookstore voucher (value CHF 150) as a compensation. Data collection was iterative in three recruitment rounds, alternating with analysis. The final sample size was established based on the principle of thematic saturation, defined as the point at which no new codes emerged, and the research team considered the main themes to be covered in sufficient detail.^22,23^

Analysis was organized on the principles of the Framework Method, which is suited for comparison of data across cases.^24^ Our mixed approach combined inductive analysis with deductive elements informed by concepts derived from our conceptual framework. All transcripts were coded by two researchers (E.P., C.L.) using Atlas.ti Windows (version 9);^25^ the first three interviews were also coded by a third researcher (D.S.) to ensure a broad perspective. Codes and emerging themes were discussed between E.P., H.M., and C.L. in batches of three to four interviews. The entire research team discussed the analysis between the second and third rounds of recruitment and after the 14 interviews to synthesize emerging patterns and discuss the findings’ meanings. Thematic saturation occurred after 12 interviews; the last two interviews confirmed the final analysis.

### Trustworthiness and Reflexivity

To enhance trustworthiness,^26^ we confronted our findings and interpretations to research conducted in other contexts^8,15,17^ and included several researcher perspectives. E.P. has a background in pediatrics and public health; primary care career choice is her main research domain. C.L. was a doctoral student to H.M. and pursuing postgraduate training in primary care at the time of the study, thus contributing a trainee perspective. C.L. and H.M. practice in France, thus contributing a transnational perspective. Other authors contributed through their experience in academic primary care (H.M., J.S., D.M.H.) and medical education (M.A., A.B.). We checked the study design and reporting for compliance with the Standards for Reporting Qualitative Research checklist.^27^

## RESULTS

**Figure 1:**
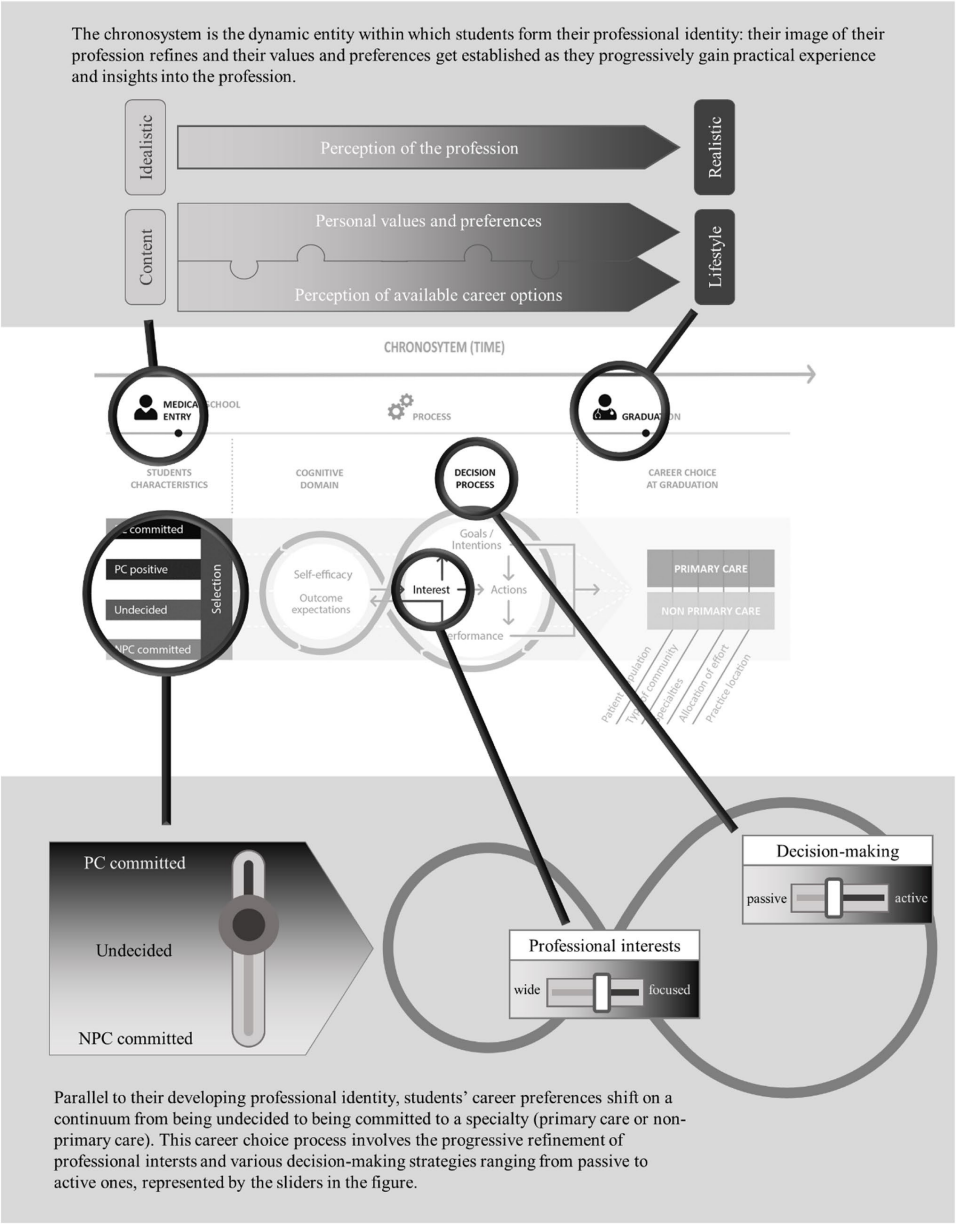
**Based on the findings of a qualitative study of career choice during medical school, concepts of a previously developed conceptual framework (Supplemental Digital **Appendix 1**) were refined to better illustrate the elements involved in the decision-making process. Part of the original framework is represented in the middle of the figure (drawn from the original publication**^7^**); elements that were refined are shown through the “magnifying glasses.”**

Table 1 presents participants’ characteristics. Themes identified in their narratives were grouped under two overarching topics (Table 2): the emergence of a professional identity, and trajectories of career-related decision-making. Figure 1 illustrates how these findings relate to the conceptual framework; illustrative quotations for each theme are provided in Table 2.

**Table 1:** Characteristics of Participants in a Qualitative Interview Study Exploring Career Choice During Undergraduate Medical Education

Participant no.*	Age at time of interview	Gender	Interview duration (minutes)	Career choice at time of interview (postgraduate training year 1)	Career choice trajectory predicted from quantitative survey†
01	26	M	43	Non-primary care (gynecology/obstetrics or dermatology)	Undecided
02	26	F	38	Primary care (adult)	Primary care–committed
03	23	F	47	Non-primary care (internal medicine subspecialty)	Undecided
04	26	F	47	Primary care (pediatric)	Primary care–positive
05	23	F	39	Non-primary care (gynecology/obstetrics)	Primary care–positive
06	28	M	54	Primary care (adult)	Primary care–positive
07	27	F	41	Primary care (adult)	Primary care–committed
08	26	F	56	Non-primary care (pediatric subspecialty)	Primary care–positive
09	26	F	45	Non-primary care (hospital internal medicine)	Primary care–positive
10	27	F	42	Non-primary care (oncology)	Primary care–positive
11	25	M	49	Primary care (adult)	Primary care–positive
12	25	M	47	Primary care (pediatric)	Primary care–positive
13	25	F	26	Primary care (pediatric)	Primary care–positive
14	25	F	34	Non-primary care (neurology)	Primary care–positive

^*^Participants were numbered consecutively according to interview sequence

^†^Participants were recruited based on career intention data collected in a previous quantitative cohort study. Primary care–committed = intention to practice primary care at least three times during medical school, including the 6th year. Primary care–positive = intention to practice primary care at least once during medical school, but without fulfilling criteria for primary care–committed. Undecided = being undecided about career intention in all years

**Table 2:** Categories and Themes Identified in a Qualitative Study on Medical Students’ Career Choice, and Associated Illustrative Quotations

Overarching topics	Themes	Dimensions within themes/sub-themes	Illustrative quotations (participant number)
Emerging professional identity	Developing image of future profession (“me as a future physician”)	Image evolving from vague to concrete	*When I started, I imagined the doctor’s work much more general, and afterwards I realized that there were many specialties and subspecialties, that there was no such thing as a doctor who does a little bit of everything. Not the way I imagined it. (P7)*
		Initially idealistic image becoming realistic • Sometimes with a touch of disillusion	*I think that all first-year students... we idealize the profession a little, it’s not a kind of hero, but here’s the doctor who [...] always has an answer to everything, and the prestige that goes with it, too. (P12)* *I realized that I had idealized the profession a lot, both in terms of workload and knowledge to be acquired, and that it was complicated. It was not as obvious as I had imagined. (P3)*
	Evolving values and preferences	Motives for studying medicine: • Scientific interest • Desire to work with people	*In high school […] I really liked biology, chemistry, how the human body works in general. […] I was curious about many things, and my curiosity was drawn to medicine, I was curious to understand and learn that. (P8)* *I did not imagine myself only in chemistry, only in biology, only in physics. I thought it was too theoretical and not enough contact with practice and life, I mean with human beings. (P2)*
		Fundamental professional values: • Relationships with patients • Continuity of care • Variety of patients and conditions • Knowledge • Desire to help patients	*I made it to the second year, and it was during these first contacts with patients that I realized that this was really where I wanted to be, it was the profession that I wanted. (P9)* *I like the follow-up aspect. To follow-up on my patients to find out if they are doing well or not, the impact of treatment, to talk to them. That’s what I want to experience. (P6)* *If I were to do only one discipline my whole life, I would need a real passion, which I haven’t really found. So that’s why I want to have more diversity, to counterbalance the fact that there is not one specific thing I want to do more than another. (P11)* *What really interests me is knowledge. I mean, there are people who know everything about a domain, and this inspired me. I wanted to be like that, to really master a specific field. (P3)* *We spend time with the patients every day, we see them evolve, we see them when they arrive and when they are discharged, and this is very valuable, because we hope that when they are discharged, they are back on their feet, they are doing better, they are satisfied, so this work is quite rewarding. (P9)*
		Emerging priorities: • Work-life balance • Workplace atmosphere	*I don’t want to sacrifice my work for my personal life, and I don’t want to sacrifice my personal life for my work. Yes, the question of work-life balance is important. Because otherwise I don’t think I’m going to be good at either. (P10)* *Quality of life is how residents are treated, how women are treated in this environment. I mean, there are certain specialties that interest me a lot, but in practice, they don’t reflect what I aspire to in medicine. (P13)*
	Image of available career options	Initial vague idea about different specialties becoming clearer over time	*During the years when we were studying, from first to third year, everything was quite interesting. We had some preferences, but there was no concrete picture of what it was like in real life, in everyday life. (P10)*
		Intrinsic specialty characteristics	*One of the aspects was that I really liked seeing the variety that the discipline offers. And I also liked seeing the aspect of the ambulatory consultations, which I like much more than being at the patient’s bedside. (P1)*
		Role models	*I’m also very touched by the fact that if you have passionate people who pass on their knowledge, it’s true that you always tend to... Well, I think I was with a senior physician who was passionate and who was a great physician. As a result, it was really very interesting. (P9)*
		Working conditions in the specialty	*I always liked surgery very much, but at the same time I didn’t see myself in it, in the sense that it’s... quite a competitive field. It’s a peculiar atmosphere. And you must dedicate yourself, do a lot of overtime to be a good surgeon, I think. I didn’t see myself investing so much time in my job. (P2)*
	Matching values and images of available options	Intuition of “finding the right fit”	*I found both aspects interesting, the practice is comprehensive, these patients need social support. So, there is not only the theoretical aspect, but also the clinical aspect, the helping, I believe. And that fits my character, my vision of medicine. (P10)* *There is also a lot of intuition to say: Wait, I feel good there and that’s where I want to go, that I feel drawn to there a little more. (P8)*
Trajectories of decision-making related to career choice	Breadth of professional interest	Wide interest	*I didn’t want to be in the mindset of choosing something too early, in the sense that afterwards I might be disappointed… [I didn’t want to have] a narrow vision in the sense that this is what I want to do and the rest I’m not interested in... So, I preferred to remain open and to find out what really interested me. (P11)*
		Focused interest	*I think it was clear that I wanted to do internal medicine. I guess I didn’t really know why I thought that, but I did think that. […] I never had the idea to become a surgeon or a pediatrician, maybe that’s why I tunneled, I told myself that I would stay in that field. (P9)*
		Periods of indecision	*I think it was obvious that I wanted to start with internal medicine. Afterwards, whether to continue in this field, I wasn’t sure, but during my studies, one of the only certainties I had, was to start with one or two years of internal medicine, and then see if there was a specialty that I preferred. (P3)*
		Awareness of impact of working conditions on interest	*I have quite broad interests, so I chose my placements in relation to what I was interested in. I can’t say that there were areas that I found less interesting, but what really made the difference was whether the team worked well or not. (P7)*
	Strategies to advance career-related decision-making	Career planning	*During medical school they don’t tell us about career planning. [I got information] by calling senior residents and saying: «I’m interested in what you’re doing. Can we have coffee together and you explain to me how it works? » (P8)*
		Confirmation of interests	*I had really liked pediatrics [in my 4th year clerkship]. I had done three weeks in [a small town]. And I decided that in my last year I would go back there to see if it confirmed my interest in pediatrics. (P2)*
		Considering alternatives	*At one point I considered doing oncology, so really something different. So I did a clerkship there in my last year. And I really liked it, but I was already hired in pediatrics, and also oncology is quite tough. I had to cope with some tough situations. So, I told myself: I think I prefer pediatrics after all. (P4)*
		Leaving doors open	*Throughout my studies, and even now, I’ve never put any pressure on myself, in the sense of: I’m going to find out what pediatrics is like, if I like it, I’ll like it, if I don’t like it, I’ll change, and really, I’m just going to take it easy. (P13)*
		Testing possibilities	*I had a preference for internal medicine, so in my last year I chose mostly medical subspecialties, cardiology, nephrology, gastroenterology… Some of these were a bit vague for me. We see these topics in lectures, but what does it look like in real life? (P11)*
		Exploration	*For a long time I didn’t know. I had interests in a little bit of everything, then I had interests in nothing, and then... […] my interests became more focused, and then I was able to sort things out a bit, and I came across something that […] corresponds well with what... I think I had to sort it all out to be able to focus more. (P10)*
		Exclusion	*It’s clear that I’ve never said to myself: I’m going to study medicine because I want to be a surgeon…. (P9)*
		Passive behaviors	*I admit that I was really hoping that something would click for me. I always said to myself: I’m waiting for the placements to see and decide, and I think I’m still waiting. I’m waiting to work at the university hospital at a specific department and to know whether this is for me or not. (P3)*

### Emerging Professional Identity

Our conceptual framework integrated the concept of time (the *chronosystem*) mainly in relation to evolving influences. In our participants’ narratives, however, the major impact of time was in relation to their self-image as future physicians, which evolved as values clarified and priorities emerged. This developing identity was continuously matched to career options, becoming more concrete as students experienced clinical work.

#### Me as a Future Physician

Participants’ image of their future profession, initially vague, became more concrete over time. Through their retrospective, critical lens, participants often mentioned having an “idealistic” image of their future profession at the beginning of medical school. This image was progressively revised with practical clerkship experiences and became more “realistic,” sometimes even with a touch of disillusion.

#### Evolving Values and Preferences

Students’ professional values, the foundation of their developing professional identity, clarified in parallel with this evolving image of the future. Scientific interest and a desire to work with people were their main motivators for choosing to study medicine. Relationships with patients and continuity of care were fundamental professional values shared by all participants and confirmed through practical experiences. Regardless of the chosen specialty, all participants emphasized the importance of encountering a variety of patients and conditions. Compared to participants training in primary care, those who were training in a non-primary care discipline associated their chosen field with specific, focused knowledge and the desire to master a discipline in all its details. Also, they placed importance on “really helping patients,” which they defined as having concrete solutions to patients’ problems and observing short-term measurable results. For these participants, primary care was often associated with psychosocial care and not being able to “really help patients.” On the other hand, primary care–oriented participants placed importance on staying in a broad field and not wanting to “over-specialize.”

The influence of working conditions emerged progressively, guided by students’ experiences in various clinical environments. Priorities were increasingly driven by anticipated future needs, and the desire for a positive work-life balance and an enjoyable workplace atmosphere became decisive for career choice. Participants described increasingly projecting into the future, in which spending time with family and extraprofessional activities became paramount. A positive work-life balance was often mentioned as the main motivator for choosing primary care.

#### Image of Available Career Options

Students continuously matched their emerging professional identity and evolving priorities to their perceptions of available careers. During pre-clinical years, students only had vague ideas about career possibilities and described the curriculum as theoretical and lacking connections with their future profession. Later, clinical clerkships were the main source of knowledge about different careers. Specialties’ intrinsic characteristics, such as the types of patients and professional activities, determined their interest in the specialty by matching these characteristics to their values of variety, continuity of care, knowledge, and helping patients. Role models reflected students’ values of teaching and knowledge, relationship with patients, and positive work-life balance. Students became increasingly aware about working conditions in different specialties. Work environment and team atmosphere were attributed more weight than the specialties’ intrinsic characteristics in the decision-making process. Ultimately, it was often an intuition of having found the “right fit” that determined matching to a career option, in terms of personality and the feeling of belonging; work-life balance and anticipation of personal satisfaction were important elements in this process.

### Trajectories of Decision-making Related to Career Choice

One of our framework’s assumptions was that students would follow distinct trajectories regarding career intentions. We hypothesized that primary care–positive individuals would have more stable and focused career intentions, but it remained unknown when these intentions would become stable and how this would translate in terms of exploration of other career options or of career planning strategies. Overall, our participants’ narratives matched their trajectories predicted by the survey data that informed sampling. However, our analysis revealed that reality was more nuanced than the anticipated distinctive trajectories.

#### Breadth of Professional Interests

As students’ professional identities emerged and career options clarified, they started to pinpoint their professional interests and focus on specific career options. This can be illustrated by the metaphor of photographers taking a picture of a landscape: Whereas some use a wide-angle lens to capture various parts of the landscape, others use a telephoto lens to zoom in on a specific aspect. Translated to our study findings, this means that some students’ interest stayed large (“wide-angle lens”), without wanting to exclude certain options too soon and allowing for flexibility in their choice process. Other students’ interest focused on a specific domain early on (“telephoto lens”), excluding other career options from the outset. Some students described periods of indecision, during which they kept a wide perspective on possibilities while also trying to find a point of focus for their professional interests.

#### Strategies to Advance Career-Related Decision-making

**Figure 2:**
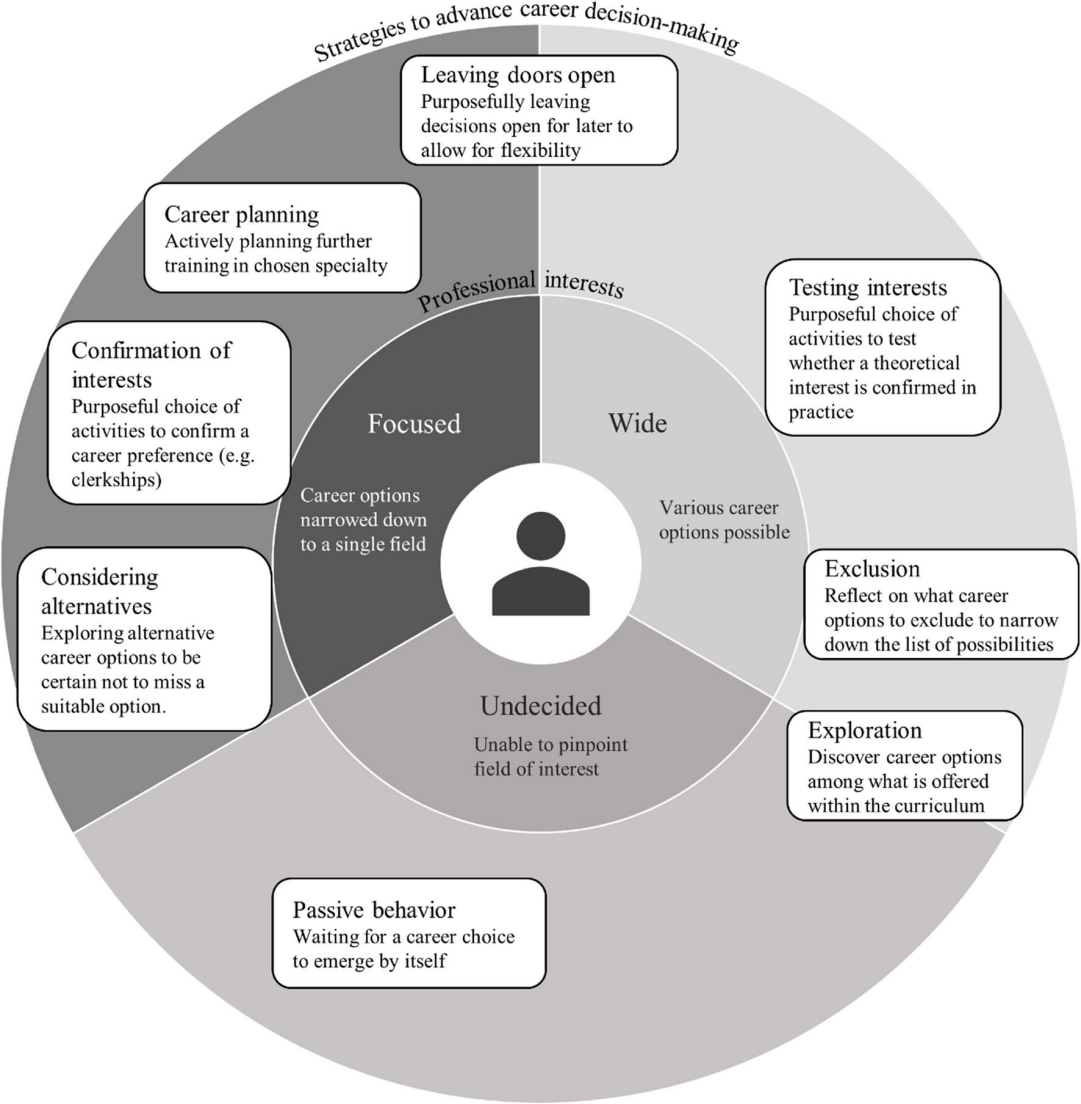
**This figure summarizes medical students’ strategies to advance career-related decision-making: Depending on the breadth of professional interests, students apply different strategies to advance career-related decision-making and career planning. This schematic representation may be useful to determine a student’s current situation within the career choice process and may help assisting them with career-related decision-making.**

Students employed various strategies to narrow down their professional interests and advance the decision-making process (Fig. 2). Our framework suggested that students’ decision-making was mostly an active and structured process, in which actions were driven by goals and led to outcomes. In our study, such active strategies were mostly used by students who were already focused on a specific field: They actively confirmed their interest (e.g., by purposefully choosing clerkships) and/or actively planned their career by arranging postgraduate training and engaging in becoming part of the community of their chosen field. Some of these students also actively explored alternative career options with the aim of reaffirming their first choice and to be certain not to miss a suitable career option.

Students with wider interests tended to explore available options, e.g., by choosing clerkships in many different fields. Emerging interests were then tested through further clerkships, which allowed students to experience the career option in “real life.” Interests were often narrowed down by exclusion: Students seemed to find it easier to start the decision-making process by identifying elements that clearly did not match their interests or priorities (e.g., excluding surgical fields from the list of possibilities). Many participants went through periods of indecision during which they adopted more passive attitudes, such as waiting for a choice to “appear” by itself. During these periods, clerkships offered during clinical training were opportunities to discover various fields, often leading to a refinement of interests and priorities.

Most participants engaged in several of these behaviors, either concomitantly or successively, depending on the stage of development of their career focus. This process of finding a focused interest is intimately intertwined with the elements of the developing professional identity described above. For example, the idealistic-to-realistic concept can be interpreted as being one facet of narrowing down professional interests, corresponding to a more precise and “real-life” image of their professional future. At the same time, evolving priorities and the increasing projection into the future lead students to shift their focus on specialties that best answer these needs.

## DISCUSSION

The main insight from this exploration of medical students’ career choice is the overarching role of developing professional identity. As students gained practical experience and observed physicians’ daily work, their initially unprecise and idealistic image became concrete and realistic. This was expressed as changing priorities: content-based considerations (about *what* they wanted to be) were superseded by lifestyle-based preferences about *how* they wanted to work. To navigate through this process, students applied various strategies depending on the breadth of career interests, aiming to confirm interests, seek alternatives, or explore various options.

We found that our conceptual framework portrays the main elements involved in career decision-making; however, we revised some elements based on our findings (Fig. 1). The “chronosystem” was initially understood as a longitudinal element of changing influences on students’ career decision-making. Our findings suggest that the developing professional identity forms the chronosystem’s overarching element, affecting the entire career choice process in which values evolve, priorities emerge, and practical experiences inform decisions. This process represents an essential task of the developmental stage of emerging adulthood, characterized by change and exploration of a variety of life directions and involving discovery of a professional identity.^28,29^

Our framework’s anticipated career choice trajectories are to some extent grounded in real life; however, our study emphasizes that they are not as clear cut and are better described as a continuum of commitment to career options, as suggested in a recent quantitative study.^14^ Our participants’ breadth of professional interest and the resulting decision-making strategies, ranging from active to passive, determined individual career choice trajectories.

Medical students’ career choice has traditionally been described as a matching process between personal interests and perceived specialty characteristics.^30^ Our findings refine this concept by suggesting that matching is progressive and includes intuition: Students describe a sense of fitting to a career when they perceive that their personality matches the specialty and its associated lifestyle. To reach this point, students need to progress through different stages of medical education and continuously process their observations to evaluate which option best responds to their emerging priorities.

The shift from content-based to lifestyle-based preferences was the most salient aspect of professional identity development, confirming previous observations.^8,17,31,32^ This has been attributed to shifting priorities in the millennial generation.^33–35^ A recent study suggested changing preferences to be related to personality changes during medical school;^8^ however, our findings suggest that these observations may rather be due to the emergence of a coherent and permanent set of values and the discovery of the profession.

Cuesta-Briand and colleagues identified two main trajectories in young physicians’ career decision-making in their recent qualitative study:^17^ “planners”—students committed to a career and proactively engaging in career planning—versus “explorers”—students less committed, exploring and eliminating options, and keeping an open mind. Our study suggests that most students engage in activities related to both exploration and planning, depending on the stage in their career choice process. Many participants continued exploring even when committed to a career, because they did not want to risk “missing” an alternative option.

This role of exploratory behavior further stresses the importance of exposure to a variety of clinical environments, the impact of which on career choice has previously been highlighted.^5,6,9^ Besides helping students refine their interests in terms of specialty content, clerkships have a profound impact on career choice by allowing students to observe the working environment. This interplay between personal development and educational environment has an extensive theoretical basis in the vocational psychology literature. By engaging in diverse work-related activities, individuals discover their abilities and interests, build self-efficacy beliefs, and reflect on their priorities, leading to the development of a professional identity.^33,36^

### Strengths and Limitations

Our study extends former qualitative research by exploring career choice as a developmental process. We did not apply a truly longitudinal approach, as our findings represent participants’ recollections of the past; they may have been different had we conducted multiple interviews with each participant. Through our approach, we collected individuals’ stories of career decision-making, rather than a series of factual accounts;^37^ participants thus linked past experiences to their current professional identity, making sense of how certain events fit into their career choice narrative.^33^ Another strength is the link with a previous cohort study allowing for purposive sampling to include participants with different degrees of interest in primary care, thus providing a varied yet focused sample.

We acknowledge the limited transferability of our findings as the study was conducted in a single context and specific population. We focused our analysis on broad processes related to career decision-making, and triangulation with research conducted in other countries revealed similarities. Also, we were mindful of researcher perspectives influencing data collection and analysis, managing this issue through an iterative process of data collection and repeated discussions in the team, combining various backgrounds and enriched by viewpoints from a neighboring country with a different training system.

Our choice of the study population was guided by our aim to better understand primary care career choice, thus excluding individuals who never expressed an interest in primary care. Although primary care career choice likely has many aspects in common with career choice in general, our study design does not allow us to draw firm conclusions about other specialty choices. Notably, work-life balance was cited as one of the principal motivators for becoming a primary care physician, which is in line with previous studies on primary care career choice.^38–40^ It would be interesting to explore in more detail whether work-life balance is a similar motivator for students interested in other specialties.

### Implications and Avenues for Further Research

Our insights suggest several avenues for increasing the attractiveness of primary care during medical school and thus encouraging students to pursue postgraduate training in this field. Most importantly, the curriculum should be aligned with students’ developing professional identity and associated needs. For example, the motivation for relationships with patients should be taken up by the curriculum from the beginning and continuously reinforced. Later, clerkships should integrate elements of successfully balancing work and private life. Besides adapting the curriculum, structured career counseling programs are recommended to support students in this potentially stressful task.^41,42^ We suggest that physicians solicited to give career-related advice assist students identify their values and priorities and match these to the professional context. Career support should always discuss individual work-life balance issues, regardless of the students’ situation.^43^

Our findings also suggest future research directions. Clerkships seem to be an important driver of the affective component of self-efficacy, illustrated by the importance that students attribute to a positive atmosphere and teamwork during clerkships. Other authors have called for further research about what makes a positive and negative clinical experience;^8^ our findings endorse these calls and suggest that affective aspects of clerkships should be explored in more detail.

Our framework includes elements of social cognitive career theory, which posits that people make decisional progress when engaging actively in decisional behaviors;^44^ thus, ample opportunities for career exploration are key. However, our findings highlight that students may also take a more passive stance, resulting in different needs. Exploring students’ role in their career decision-making with a specific perspective on the active versus passive spectrum of behaviors might provide further insights about how to effectively support students in their career choice.

## CONCLUSIONS

Our narrative approach revealed participants’ stories about developing professional identities from a vague, idealistic to a concrete, realistic image, resulting from continuous interaction between personal development and learning about their future work environment. This dynamic process overarches career-related decision-making, resonating with our conceptual framework’s chronosystem. In their efforts to make primary care careers more attractive, medical schools should remember that career choice happens during a life stage characterized by exploration and cognitive maturation. Personal experiences and observations of teamwork and working conditions during clinical encounters are crucial in shaping this path. Offering sufficient primary care exposure in a safe and individualized environment allows students to clarify their values, prioritize their needs, and explore various facets of their future professional activity, and will likely support more students in choosing a primary care career in the future.

### Supplementary Information

Below is the link to the electronic supplementary material.

Supplementary file1 (PDF 158 KB)

Supplementary file2 (PDF 90 KB)

Supplementary file3 (PDF 149 KB)

## Data Availability

The datasets generated and analyzed during the current study are available from the corresponding author on reasonable request.
