# Supplementary material for: Dynamics of Students’ Career Choice: a Conceptual Framework–Based Qualitative Analysis Focusing on Primary Care
Source: J Gen Intern Med. 2023 Dec 15;39(9):1544–55. doi: 10.1007/s11606-023-08567-9 (PMC11254893; doi:10.1007/s11606-023-08567-9)

Supplemental Digital Appendix 1: Original figure of a conceptual framework of medical students' primary care career choice

Students enter the process of decision making with their personal characteristics and initial interest in primary care, and they emerge at graduation with a choice for their future career (depicted in the central part of the framework). This process is influenced by various factors, which are grouped into hierarchical systems (depicted in the outer part of the framework as concentric circles). The lower part of the figure is a detailed representation of students' career choice process. Preexisting personal characteristics influence students' initial interest in primary care; based on this interest, students fit into one of four distinct groups - primary care (PC) committed, primary care positive, undecided, and non-primary-care (NPC) committed. During this process, students may change career paths, and then they make a career choice at graduation regarding their postgraduate training.

Figure reproduced with permission from: Pfarrwaller E, Audétat M-C-, Sommer J, et al. An expanded conceptual framework of medical students' primary care career choice. Acad Med 92(11):1536-1542, November 2017.

doi: 10.1097/ACM.0000000000001676

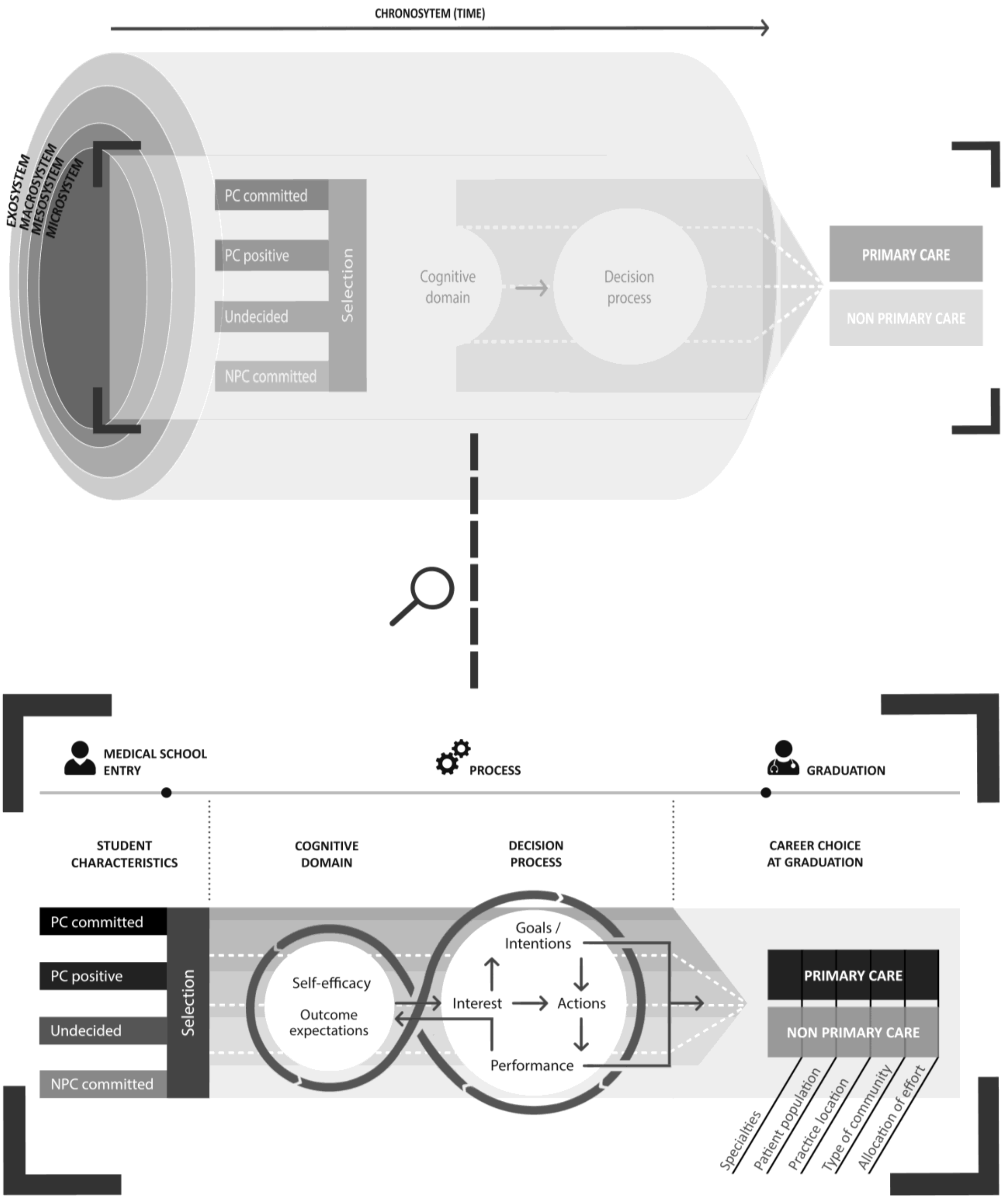

Supplement: Supplementary file 1 — Supplementary file1 (PDF 158 KB) [file 11606_2023_8567_MOESM1_ESM.pdf]
