# Supplementary material for: Dynamics of Students’ Career Choice: a Conceptual Framework–Based Qualitative Analysis Focusing on Primary Care
Source: J Gen Intern Med. 2023 Dec 15;39(9):1544–55. doi: 10.1007/s11606-023-08567-9 (PMC11254893; doi:10.1007/s11606-023-08567-9)

Appendix 2: Detailed description of participant sampling for a qualitative study to explore career decision-making during medical school

In the present study, we chose to focus on career choice related to primary care. Thus, we applied a purposive sampling strategy to identify individuals with varying degrees of interest in primary care careers. We based our sampling on data collected in a quantitative cohort study coordinated by MA, that included yearly collection of students' career intentions. The cohort study recruited students in their first year of medical school and followed them up with a yearly questionnaire. After the last questionnaire (at the end of the final year of medical school), participants provided their e-mail address and consented to being contacted again for further studies. For our qualitative study, we focused on first-year postgraduate trainees who had expressed an interest in primary care at some point during medical school. We applied a coordinated multi-step sampling approach (Figure): EP defined the baseline population from anonymous survey data. MA (the cohort study coordinator who had access to the de-anonymized version of the questionnaire data) selected groups of individuals to be contacted iteratively with the aim of providing a balanced and varied sample in terms of career preferences and gender. She then forwarded their contact details (name and e-mail address) to EP, who invited potential participants the study by e-mail in three iterative recruitment rounds.

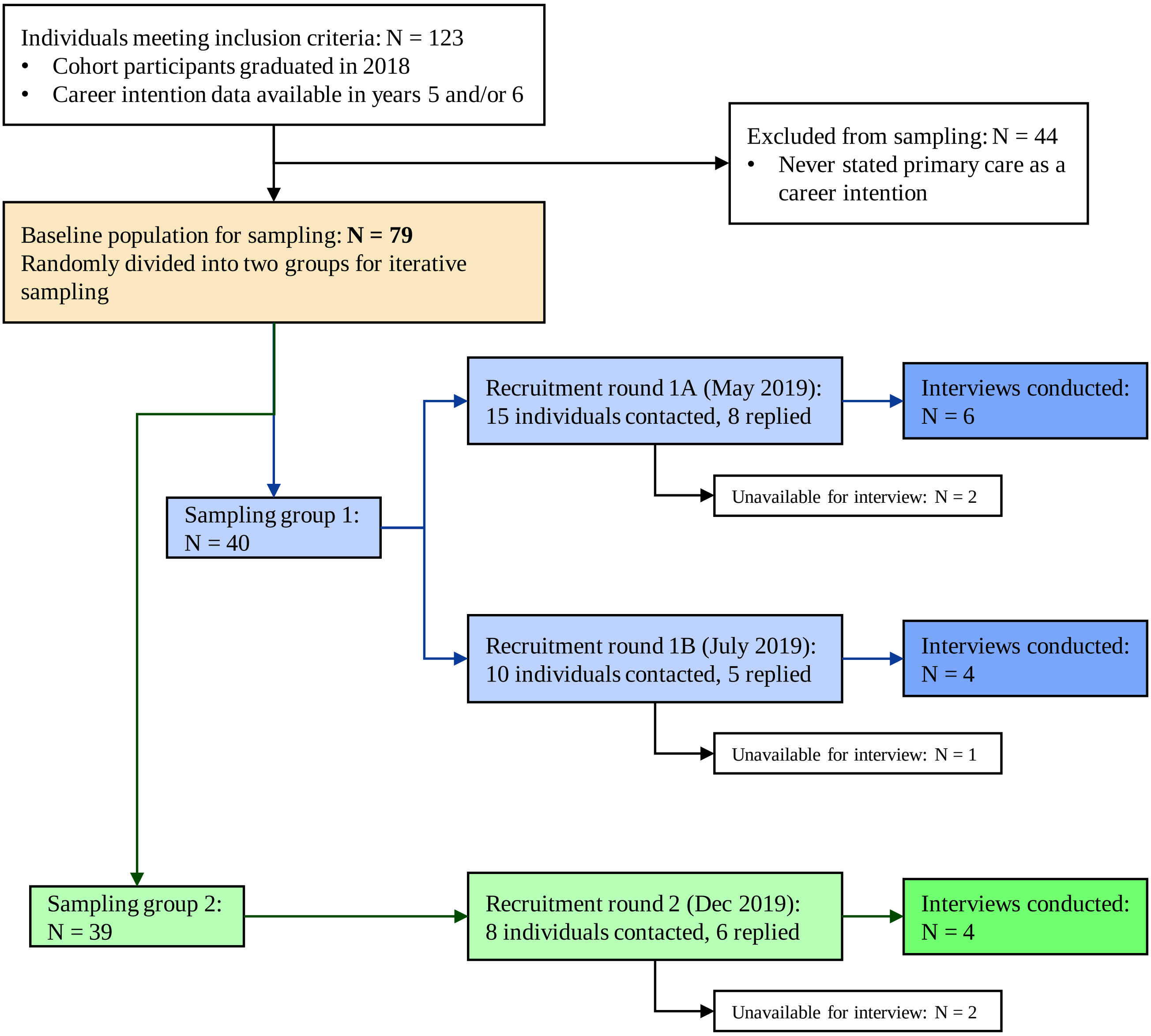

Supplement: Supplementary file 2 — Supplementary file2 (PDF 90 KB) [file 11606_2023_8567_MOESM2_ESM.pdf]
