# Supplementary material for: Dynamics of Students’ Career Choice: a Conceptual Framework–Based Qualitative Analysis Focusing on Primary Care
Source: J Gen Intern Med. 2023 Dec 15;39(9):1544–55. doi: 10.1007/s11606-023-08567-9 (PMC11254893; doi:10.1007/s11606-023-08567-9)
Supplement: Supplementary file 3 — Supplementary file3 (PDF 149 KB) [file 11606_2023_8567_MOESM3_ESM.pdf]

Supplemental Digital Appendix 3: Interview guide for a qualitative study exploring career choice during undergraduate medical education

This document contains the original interview guide in French, followed by an English version (automatic translation by Deepl.com).

Exploration du choix de carrière pendant les études prégraduées chez les participants de la cohorte CAPA (1ère année postgraduée)

Entretiens semi-dirigés - Guide d'entretien

Instructions :

- L'entretien suivra, si possible, les phases d'entretien ci-dessous, mais l'objectif principal est de laisser le participant raconter son histoire de comment son choix de carrière s'est développé dans le temps. Il ne sera donc pas obligatoire de suivre la structure prédéterminée, avec l'exception des premières **questions ouvertes** qui seront posées à tout le monde (sauf si le participant aborde spontanément le thème).
- Le guide d'entretien détermine les **sujets à approfondir** (s'ils sont spontanément abordés par le participant) ou à aborder en deuxième partie d'entretien (si pas abordés spontanément).
- Eviter les questions de « pourquoi » et favoriser les questions encourageant des réponses descriptives. Par ex : « racontez-moi comment était ce stage », « décrivez cette personne », etc.

Page participant

Nom : Numéro d'identification :

Contact (mail, tél.) :

Checklist :

- ☐ Consentement signé
- ☐ Accord pour extraction données questionnaire : oui / non
- ☐ Accord pour être recontacté : oui / non
- ☐ Donné bon d'achat et fait signer le reçu

Page à détacher et à conserver séparément

Feuille d’entretien

Date entretien :

Numéro d’identification :

Interviewer :

Autres personnes présentes :

Données personnelles du participant :

Genre : F / M

Activité actuelle (lieu, type d’institution, spécialité) :

Notes d’observation :

Phase de l'entretien	Questions/Thèmes principaux	Précisions / Relances
**Bienvenue et information**	• Présentation (interviewer, év autre personne si présente)<br>• Informations sur l'étude<br>• Informations sur l'entretien (enregistrement, durée)<br>• Signer le consentement	Voir feuilles annexes et page participant
**Données personnelles**	Genre<br>Activité actuelle -> sert de récolte de données, mais aussi d'introduction et de « ice-breaker » car fait le lien avec la première question.	Voir feuille d'entretien
(enclencher l'enregistreur)<br>**La situation actuelle du participant :**<br>• Son activité actuelle<br>• Sa vision sur les perspectives	**Pour commencer, racontez-moi où vous en êtes actuellement dans votre formation.**	Décrivez ce que vous aimez / n'aimez pas dans votre activité actuelle
	**Comment vous envisagez la suite ?**	• Comment vous prévoyez d'arriver là ?<br>• Comment vous êtes-vous organisé pour la suite ?<br>• Qu'est-ce qui vous attire dans cette spécialité ? Est-ce qu'il y a des aspects que vous aimez moins ?<br>• Si hésitant entre 2 spécialités : qu'est-ce qui vous attire dans l'une/l'autre ? Qu'est-ce que vous aimez moins dans l'une/l'autre ?<br>• Comment est-ce que vous vous voyez dans 10 ans ?

**Le développement du choix de carrière jusqu'au moment actuel :**<br>Raconté dans les mots du participants	**Comment êtes-vous arrivé là où vous êtes actuellement ?**	**Moments charnières/transitions** :<br>• Quand avez-vous su que c'est cette spécialité que vous vouliez faire ?<br>• Racontez ce qui s'est passé pendant …<br>• Décrivez ce stage…<br>• Moments d'hésitation : quand, où, points positifs/négatifs d'une certaine spécialité
	En cas de participant toujours indécis :<br>Qu'est-ce qu'il faudrait pour que vous puissiez vous décider ?	Hésitations entre X et Y – avantages et désavantages de chaque…
	En cas de participant qui a « toujours su » ce qu'il voulait faire :<br>Est-ce qu'il y avait des moments d'hésitations ?<br>Quelle était votre image de la spécialité au début des études ? Et à la fin ?	

**Approfondir/aborder thèmes principaux**, selon le contenu des réponses libres et le flux de l'entretien<br>(pas de séquence pré-déterminée)	**Ligne de temps / longitudinalité :**<br>Couvrir toute la **durée des études**<br>• Explorer la durée et la temporalité des influences mentionnées par le participant (avant-après-maintenant…)<br>• Possibilité de s'aider avec un croquis (timeline) pour structurer	• Et avant ? (en 1ère, entre la 1ère et la 3ème, avant vos stages, etc)<br>• Comment vous voyiez ce que vous vouliez faire ?
	Parler de la **période avant les études**	• Avant de commencer vos études, quelle était votre idée de la médecine / de votre future profession ?<br>• Comment avez-vous décidé de faire des études de médecine ?
	**Personnes d'influence :**<br>Professionnels (modèles de rôle)	• Avez-vous rencontré une personne à qui vous vouliez ressembler au niveau professionnel ?<br>• Comment était cette personne ? Comment agissait-elle ?
	Conseils de carrière	• Est-ce que vous vous êtes adressé à une personne pour clarifier votre choix de spécialité ?
	Pairs	• Est-ce que vous avez parlé de vos choix de carrière avec vos collègues étudiants ?<br>• Quel effet cela a-t-il eu sur vous ?
	Autres personnes (si mentionnées par le participant)	Décrire sa fonction, comment elle était…

	**Intérêts personnels sur certains sujets** (si mentionnés par le participant) :<br>Décrivez ce qui vous intéresse/attire dans ce sujet	Conséquences de cet intérêt/attrait (par ex. stages à option, etc.) : actions, résultats
	**Eléments du processus cognitif** (cadre conceptuel) :<br>Pas de question précise, mais garder ce processus en tête durant l'entretien (**auto-efficacité**, **attentes**, boucle de feedback : **intérêts-buts-actions-résultats**)	• Est-ce que vous vous sentiez prête à …<br>• Qu'est-ce qui a fait que vous vous sentiez débordée / prête ?<br>• Qu'est-ce que vous attendiez de… ?
**Aborder la thématique de la MPR** :<br>Idées / vues spécifiques sur la MPR	**Ces dernières années on a beaucoup entendu parler qu'il y avait un manque de médecins de famille en Suisse.**<br>**Est-ce que pendant vos études vous en aviez entendu parler ?**	• Qu'est-ce que vous avez entendu ?<br>• Quel est votre point de vue ?<br>• Quel était votre point de vue pendant vos études ?<br>• Est-ce que cela vous encourage / décourage dans votre choix ?
**Fin de l'entretien**	**Résumé** de ce qui a été dit (év en s'aidant du croquis)<br>• Est-ce qu'il y a quelque chose dont on n'a pas parlé / que vous aimeriez ajouter ?<br>• Des questions ?	
**Conclusion** *(on peut arrêter l'enregistrement)*	Remercier le participant<br>Demander accord pour être recontacté :<br>• Pour relire et valider nos conclusions<br>• Pour relire les citations utilisées dans la publication<br>• Pour une éventuelle suite ou un complément de cette recherche	

Exploring career choice during pre-graduate studies among participants in the CAPA cohort (1ère post-graduate year)

Semi-structured interviews - Interview guide

Instructions :

- The interview will follow, if possible, the interview phases below, but the main objective is to let the participant tell their story of how their career choice developed over time. It will therefore not be compulsory to follow the predetermined structure, with the exception of the first **open-ended questions**, which will be put to everyone (unless the participant spontaneously broaches the subject).
- The interview guide determines the **topics to be explored in greater depth** (if spontaneously raised by the participant) or to be addressed in the second part of the interview (if not spontaneously raised).
- Avoid "why" questions and encourage descriptive answers. For example: "Tell me what this course was like", "Describe this person", etc.

Participant page

Name : Identification number :

Contact (mail, tel.) :

Checklist :

- ☐ Signed consent
- ☐ Agreement to extract questionnaire data: yes / no
- ☐ Agreement to be recontacted: yes / no
- ☐ Gave voucher and had receipt signed

Page to be detached and kept separately

Interview documentation

Interview date:

Identification number:

Interviewer:

Others present:

Participant's personal data:

Gender: F / M

Current activity (location, type of institution, specialty):

Observation notes :

Interview phase	Main questions/themes	Clarification / Follow-up
**Welcome and information**	• Presentation (interviewer, other person if present)<br>• Study information<br>• Information about the interview (recording, duration)<br>• Sign the consent form	See attached sheets and participant page
**Personal data**	Type<br>Current activity -> serves as data collection, introduction and ice-breaker, as it links up with the first question.	See maintenance sheet
(switch on recorder)<br>**The participant's current situation :**<br>• Current activity<br>• Their vision of the future	**To begin with, tell me where you are in your training.**	Describe what you like/dislike about your current activity
	**How do you see the future?**	• How do you plan to get there?<br>• How did you get organized for the next stage?<br>• What attracts you to this specialty? Are there any aspects you dislike?<br>• If you're hesitating between 2 specialties: what attracts you to one or the other? What do you like less about one or the other?<br>• How do you see yourself in 10 years?
	**How did you get to where you are today?**	**Turning points/transitions** :

**The development of the career choice up to the present time :**<br>Told in the words of the participants		• When did you know this was the specialty you wanted to pursue?<br>• Tell us what happened during ...<br>• Describe this internship...<br>• Moments of hesitation: when, where, positive/negative points of a certain specialty
	If the participant is still undecided :<br>What would it take for you to make up your mind?	Hesitations between X and Y - advantages and disadvantages of each...
	For participants who "always knew" what they wanted to do:<br>Were there moments of hesitation?<br>What was your image of the specialty at the start of your studies? And at the end?	
	**Timeline / longitudinality :**	

**Deepen/address main themes**, depending on the content of free responses and the flow of the interview<br>(no pre-determined sequence)	Cover the entire **study period**<br>• Explore the duration and temporality of the influences mentioned by the participant (before-after-now…)<br>• You can use a timeline to help structure your project.	• And before? (in 1ère , between 1ère and 3ème , before your internships, etc)<br>• How did you see what you wanted to do?
	Talking about the **period before studying**	• Before starting your studies, what was your idea of medicine / your future profession?<br>• How did you decide to study medicine?
	**Influential people :**<br>Professionals (role models)	• Have you met someone you wanted to be like professionally?<br>• What was this person like? How did she act?
	Career advice	• Did you ask anyone to clarify your choice of specialty?
	Peers	• Have you discussed your career choices with your fellow students?<br>• What effect did this have on you?
	Other persons (if mentioned by participant)	Describe her function, what she was like…
	**Personal interests on certain subjects** (if mentioned by the participant) :<br>Describe what interests/attracts you about this topic	Consequences of this interest/attraction (e.g. electives, etc.): actions, results

	**Elements of the cognitive process** (conceptual framework) :<br>No specific questions, but keep this process in mind during the interview (**self-efficacy**, **expectations**, feedback loop: **interest-goals-actions-results**).	• Did you feel ready to ...<br>• What made you feel overwhelmed/ready?<br>• What did you expect from...?
**Addressing the theme of PRM**: Specific ideas / views on PRM	**In recent years, we've heard a lot about the lack of family doctors in Switzerland.**<br>**Did you hear about it during your studies?**	• What did you hear?<br>• What's your point of view?<br>• What was your point of view during your studies?<br>• Does this encourage or discourage you in your choice?
**End of interview**	**Summary** of what has been said (using the sketch as a guide)<br>• Is there anything we haven't talked about / that you'd like to add?<br>• Any questions?	
**Conclusion** *(recording can be stopped)*	Thanking the participant<br>Request agreement to be recontacted :<br>• To reread and validate our conclusions<br>• To reread the quotations used in the publication<br>• For a possible continuation or complement of this research	
